# Comparing humoral immune response to SARS‐CoV2 vaccines in people with multiple sclerosis and healthy controls: An Austrian prospective multicenter cohort study

**DOI:** 10.1111/ene.15265

**Published:** 2022-02-09

**Authors:** Gabriel Bsteh, Harald Hegen, Gerhard Traxler, Nik Krajnc, Fritz Leutmezer, Franziska Di Pauli, Barbara Kornek, Paulus Rommer, Gudrun Zulehner, Sophie Dürauer, Angelika Bauer, Sarah Kratzwald, Sigrid Klotz, Michael Winklehner, Florian Deisenhammer, Michael Guger, Romana Höftberger, Thomas Berger

**Affiliations:** ^1^ 27271 Department of Neurology Medical University of Vienna Vienna Austria; ^2^ 27280 Department of Neurology Medical University of Innsbruck Innsbruck Austria; ^3^ Department of Neurology 2 Med Campus III Kepler University Hospital GmbH Linz Austria; ^4^ Medical Faculty Johannes Kepler University Linz Linz Austria; ^5^ 27271 Division of Neuropathology and Neurochemistry Department of Neurology Medical University of Vienna Vienna Austria; ^6^ 27266 Department of Neurology Pyhrn‐Eisenwurzen Hospital Steyr Steyr Austria

**Keywords:** COVID‐19, multiple sclerosis, response, SARS‐CoV‐2, vaccination

## Abstract

**Background and purpose:**

SARS‐CoV2 vaccination is recommended for patients with multiple sclerosis (pwMS), but response may be limited by disease‐modifying‐treatments (DMTs). The aim of this study was to compare the rates of humoral immune response and safety of SARS‐CoV‐2 vaccines in pwMS and healthy controls (HCs).

**Methods:**

In this multicenter prospective study on 456 pwMS and 116 HCs, SARS‐CoV‐2‐IgG response was measured 3 months after the first vaccine dose. The primary endpoint was defined as proportion of patients developing antibodies (seroconversion). Secondary endpoints included antibody level, safety and efficacy.

**Results:**

Compared to 97.4% in HCs, seroconversion occurred in 96.7% (88/91) untreated pwMS, 97.1% of patients (135/139) on immunomodulatory DMTs and 61.1% (138/226; *p* < 0.001) on immunosuppressive DMTs. Seroconversion was lowest in patients on antiCD20 monoclonal antibodies (CD20 mAbs; 52.6%) followed by sphingosine‐1‐phosphate‐receptor‐modulators (S1PMs; 63.6%). In the S1PM subgroup, seroconversion increased with lymphocyte count (odds ratio [OR] 1.31 per 0.1 G/L; *p* = 0.035). In pwMS on CD20 mAbs, B‐cell depletion decreased seroconversion (OR 0.52; *p* = 0.038), whereas time since last DMT did not. Safety of SARS‐CoV‐2 vaccines in pwMS was excellent.

**Conclusions:**

Humoral response to SARS‐CoV2 vaccines in pwMS is generally excellent. While reduced by immunosuppressive DMTs, most importantly by B‐cell‐depleting CD20 mAbs and S1PMs, seroconversion is still expected in the majority of patients. SARS‐CoV2 vaccination should be offered to every MS patient.

## Graphical abstract

Seroconversion and antibody levels after SARS‐CoV2 vaccination were comparable to healthy controls, in untreated patients with MS or MS patients on immunomodulatory DMTs, but significantly reduced (61.1%) on immunosuppressive DMTs (p<0.001).

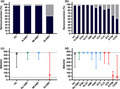

## INTRODUCTION

An unprecedented worldwide scientific effort has yielded several vaccines against SARS‐CoV‐2, for the first time relying on the concepts of mRNA (messenger ribonucleic acid) vaccination or adenovirus vector‐based vaccination [1].

Patients with multiple sclerosis (MS) are frequently treated with disease‐modifying therapies (DMTs) that interfere with the immune system, possibly limiting immune response to vaccination and the extent of protection achieved or altering the side effect profile [2, 3]. With most available DMTs (dimethyl fumarate, glatiramer acetate, interferon‐beta preparations, natalizumab and teriflunomide), adequate vaccine protection is assumed. In contrast, anti‐CD20 monoclonal antibodies (CD20 mAbs; ocrelizumab, ofatumumab, rituximab) or sphingosine‐1‐phosphate receptor modulators (S1PMs; fingolimod, ozanimod, ponesimod or siponimod) may significantly decrease vaccine response [4, 5, 6].

Here, we investigated humoral response and the adverse event profile of SARS‐CoV‐2 vaccination in patients with MS compared to healthy individuals, as well as the role of DMTs.

## METHODS

We conducted a multicenter (Vienna, Innsbruck and Linz) prospective observational study including 500 MS patients and 130 healthy controls (HCs) willing to be vaccinated against SARS‐CoV‐2. MS patients were subgrouped according to DMT status at the time of vaccination and the supposed impact of the respective DMT on vaccine response, based on its respective mechanism of action, as either untreated (N‐DMT), treated with immunomodulatory DMTs (IM‐DMTs: dimethyl fumarate, glatiramer acetate, interferon‐beta preparations, natalizumab, teriflunomide) or treated with immunosuppressive DMTs (IS‐DMTs: alemtuzumab, cladribine, CD20 mAbs, S1PMs) [3]. Based on power calculations (alpha 0.05; beta 0.80; assumed drop‐out rate 10%), we recruited 130 HCs, and 100 patients to the N‐DMT group, 150 to the IM‐DMT group and 250 to the IS‐DMT group.

Inclusion criteria for the MS group were age ≥18 years and a diagnosis of MS according to the 2017 version of the McDonald criteria [7]. Exclusion criteria comprised, among others, history of prior SARS‐CoV‐2 infection (assessed by confirmed positive SARS‐CoV2 PCR test), another autoimmune disease other than MS and Hashimoto's disease, and treatment with an immunomodulatory or immunosuppressive agent for a reason other than MS.

The primary endpoint was the proportion of patients developing antibodies against SARS‐CoV‐2 (seroconversion). Secondary endpoints included antibody levels and safety variables (local or systemic adverse events, severe adverse events).

Venous blood samples were drawn within 2 weeks before and 3 months after the first vaccination (≥3 weeks after completion of the respective vaccination regimen). Antibody testing was performed centrally by the commercially available Anti‐SARS‐CoV‐2‐QuantiVac‐ELISA (IgG; Euroimmun, Lübeck, Germany), with results shown in standardized binding antibody units per milliliter (BAU/ml). The allowed antibody level ranged from 3.2 to 384 BAU/ml, and 35.2 BAU/ml was used as the cut‐off for positive samples.

Statistical analyses were performed using SPSS 26.0 (SPSS Inc.). After univariate group comparisons, predictors of seroconversion were investigated by multivariable logistic regression analyses, with seroconversion as the dependent variable and DMT group as the independent variable, and age, sex, disease duration, time interval to last DMT intake, absolute lymphocyte count and complete B‐cell depletion (defined as <1 CD19‐positive cells/ml) as covariates. A‐priori‐defined subgroup analyses were conducted for mRNA and vector vaccines as well as in the subgroups of patients on S1PMs and CD20 mAbs using otherwise the same covariates and adjusting *p* values for multiple comparisons using the Bonferroni method. A‐priori‐defined sensitivity analyses were conducted for each DMT.

The study was approved by the ethics committees of the Medical Universities of Vienna, Innsbruck and Linz (EK Nr: 1029/2021). Written informed consent was obtained from all study participants. Data supporting the findings of this study are available from the corresponding author upon reasonable request and upon approval by the ethics committee of the Medical University Vienna.

## RESULTS

The study was completed by 116/130 HCs and by 91/100 patients in the N‐DMT group, 139/150 patients in the IM‐DMT group and 226/250 patients in the IS‐DMT group. Nine patients (HC group: *n* = 2; N‐DMT group: *n* = 2; IM‐DMT group: *n* = 2; IS‐DMT group: *n* = 3) were excluded for SARS‐CoV‐2‐antibody positivity before vaccination, 23 patients (*n* = 7/2/5/9, respectively) elected not to be vaccinated and 26 patients (*n* = 5/5/4/12, respectively) were lost to follow‐up (Table 1).

**TABLE 1:** Characteristics of the study cohort

*N* = 572	Healthy controls (*n* = 116)	Multiple sclerosis (*n* = 456)		
		No DMT (*n* = 91)	IM‐DMT (*n* = 139)	IS‐DMT (*n* = 226)
Female^a^	82 (70.7)	66 (72.5)	92 (66.2)	156 (69.0)
Age, years^b^	41.4 (12.3)	46.0 (13.7)	37.4 (10.2)	40.3 (11.0)
Disease duration, years^b^	NA	9.8 (8.1)	7.1 (6.1)	10.4 (7.9)
Disease course^a^				
Relapsing MS	NA	47 (51.6)	113 (81.3)	162 (71.7)
Primary progressive MS	NA	21 (23.1)	0 (0)	41 (18.1)
Secondary progressive MS	NA	23 (25.3)	26 (18.7)	23 (10.2)
EDSS^c^	NA	2.0 (0–8.5)	1.0 (0–7.5)	2.5 (0–7.5)
On DMT at vaccination^a^	NA	0 (0)	139 (100)	226 (100)
Time on DMT at vaccination, years^c^	NA	NA	2.6 (0–17)	2.4 (0–15)
IM‐DMT^a^				
Dimethyl fumarate	NA	NA	63 (45.3)	NA
Glatiramer acetate	NA	NA	20 (14.4)	NA
Interferon‐beta	NA	NA	22 (15.8)	NA
Natalizumab	NA	NA	24 (17.3)	NA
Teriflunomide	NA	NA	10 (7.2)	NA
IS‐DMT^a^				
Alemtuzumab	NA	NA	NA	8 (3.5)
CD20 mAbs	NA	NA	NA	116 (51.3)
Cladribine	NA	NA	NA	25 (11.1)
S1PMs	NA	NA	NA	77 (34.1)
Lymphopenia before vaccination^a^	0 (0)	1 (1.1)	43 (30.9)	84 (37.2)
Grade 3 or higher^a^	0 (0)	0 (0)	3 (2.2)	15 (6.6)
Type of vaccination^a^				
mRNA vaccine	36 (31.0)	64 (70.3)	119 (85.6)	183 (81.0)
Vector‐based vaccine	80 (69.0)	27 (29.7)	20 (14.4)	43 (19.0)

Abbreviations: CD20 mAbs, anti‐cluster of differentiation 20 monoclonal antibodies (ocrelizumab or rituximab); DMT, disease‐modifying treatment; EDSS, Expanded Disability Status Scale; IM‐DMT, immunomodulating DMT (dimethyl fumarate, glatiramer acetate, interferon‐beta preparations, natalizumab or teriflunomide); IS‐DMT, immunosuppressive DMT (alemtuzumab, cladribine, fingolimod, ocrelizumab, ozanimod, rituximab or siponimod); MS, multiple sclerosis; S1PMs, spingosin 1 receptor modulators (fingolimod, ozanimod or siponimod).

^a^ Absolute number and percentage.

^b^ Mean and standard deviation.

^c^ Median and minimum‐maximum range.

**FIGURE 1:**
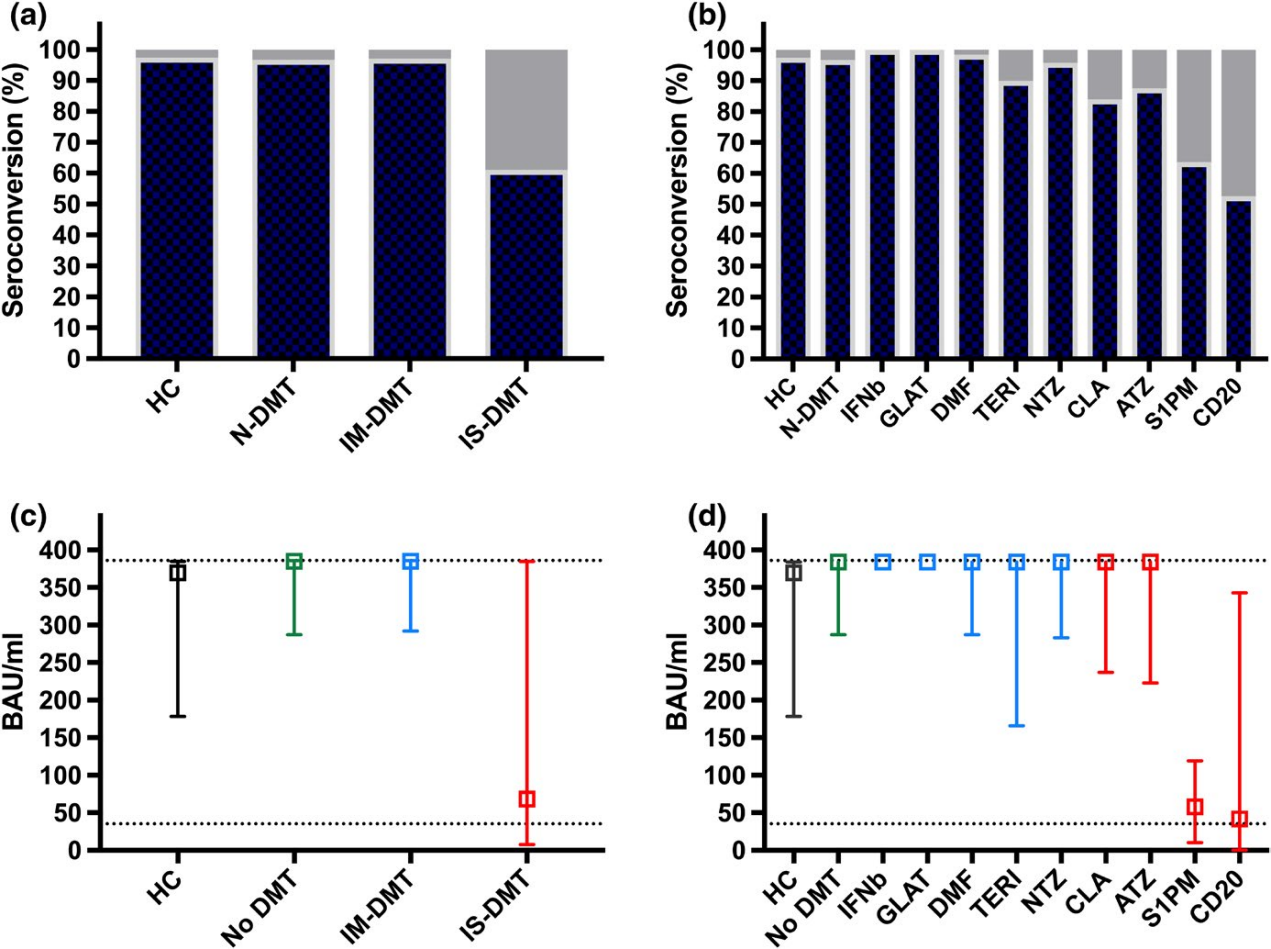
Seroconversion 3 months after SARS‐CoV‐2 vaccination differs among disease‐ modifying treatment (DMT) categories (a) and substances (b). Lower dotted line indicates the level of seropositivity and upper dotted line indicates the upper limit of antibody level at 384 BAU/ml in (c) and (d). ATZ, alemtuzumab; BAU/ml, binding antibody units per milliliter; CD20, anti‐cluster of differentiation 20 monoclonal antibodies (ocrelizumab or rituximab); CLA, cladribine; DMF, dimethyl fumarate; DMT, disease‐modifying treatment; GLAT, glatiramer acetate; IFNb, interferon‐beta preparations; IM‐DMT, immunomodulating DMT (dimethyl fumarate, glatiramer acetate, interferon‐beta preparations, natalizumab and teriflunomide); IS‐DMT, immunosuppressive DMT (alemtuzumab, cladribine, fingolimod, ocrelizumab, ozanimod, siponimod or rituximab); N‐DMT, no DMT (untreated); NTZ, natalizumab; S1PM, sphingosine 1 receptor modulators (fingolimod, ozanimod, siponimod); TERI, teriflunomide [Colour figure can be viewed at wileyonlinelibrary.com]

Seroconversion occurred in 97.4% (113/116) of HCs compared to 96.7% of patients (88/91) in the N‐DMT group, 97.1% (135/139) in the IM‐DMT group and 61.1% (138/226, *p* < 0.001) in the IS‐DMT group (Figure 1a).

Differentiating according to DMTs, we found seroconversion in 100% of patients on interferon‐beta preparations and glatiramer acetate (22/22 and 20/20, respectively), 98.4% on dimethyl fumarate (62/63), 90% on teriflunomide (9/10), 95.8% on natalizumab (23/24), 87.5% on alemtuzumab (7/8; time since last application: median 13 [range 8–31] months) and 84% on cladribine (21/25; time since last application: median 8 [range 4–19] months), which did not significantly differ compared to HCs and paitents in the N‐DMT group (Figure 1b). Patients on S1PMs (63.6%, 49/77; adjusted *p* < 0.001) and CD20 mAbs (52.6, 61/116; adjusted *p* < 0.001) showed significantly lower rates of seroconversion.

With regard to absolute antibody levels, the N‐DMT group (median 384 [interquartile range {IQR}287–384] BAU/ml) and the M‐DMT group (median 384 [IQR 292–384] BAU/ml) did not differ from the HC group (median [IQR] 370 [178–384] BAU/ml), while the IS‐DMT group had significantly lower levels (median [IQR] 68 [8–384] BAU/ml; *p* < 0.001 [Figure 1c]). Median (IQR) antibody levels were comparable to the HC and N‐DMT groups in all the DMT subgroups with the exception of the S1PM (58 [10–119] BAU/ml; adjusted *p* < 0.001) and CD20‐mAb subgroups (42 [0–343] BAU/ml; adjusted *p* < 0.001 [Figure 1d]).

In the overall cohort, the multivariable regression analysis identified IS‐DMT treatment as the only significant predictor of seroconversion (OR 0.04; *p* < 0.001), whereas neither age nor lymphocyte count nor time since last DMT intake were significantly associated with seroconversion (Table 2). In subgroup analyses, treatment with CD20 mAbs was associated with the lowest probability of seroconversion (OR 0.03), followed by S1PMs (OR 0.05) and alemtuzumab/cladribine (OR 0.18).

**TABLE 2:** Predictors of seroconversion after SARS‐CoV‐2 vaccination

Whole cohort	Seropositivity^a^		
	OR	95% CI	*p* value
Age (per 5 years)	0.99	0.96–1.01	0.271
DMT^a^			
N‐DMT	0.90	0.15–5.5	0.905
IM‐DMT	0.83	0.18–3.8	0.812
IS‐DMT	0.04	0.01–0.13	<0.001
Lymphocyte count (per 0.1 G/L)	1.14	0.88–1.59	0.234
	*R* squared 0.573; *p* < 0.001		
Subgroup analyses^b^			
S1PM subgroup			
S1PMs	0.05	0.01–0.23	<0.001
Lymphocyte count (per 0.1 G/L)	1.31	1.02–1.77	0.035
CD20 mAb subgroup			
CD20 mAbs	0.03	0.01–0.14	<0.001
Complete B‐cell depletion^c^	0.52	0.24–0.93	0.038
Time since last DMT intake (per month)	1.24	0.56–4.13	0.739
Subgroup ATZ/CLA			
ATZ/CLA	0.18	0.03–0.99	0.049
Lymphocyte count (per 0.1 G/L)	1.24	0.72–2.82	0.608
Time since last DMT intake (per month)	1.38	1.06–1.98	0.026

Abbreviations: ATZ, alemtuzumab; CLA, cladribine; CI, confidence interval; CD20 mAbs, anti‐cluster of differentiation 20 monoclonal antibodies; DMT, disease‐modifying treatment; IM‐DMT, immunomodulating DMT; IS‐DMT, immunosuppressive DMT; MS, multiple sclerosis; N‐DMT, untreated; OR, odds ratio; S1PMs, sphingosin 1 receptor modulators.

^a^ Reference category: healthy controls.

^b^ Predefined subgroup analyses of patients on S1PMs, CD20 mAbs and the combined group of ATZ and CLA vs. N‐DMT as the reference category, calculated by multivariable binary logistic regression models with seroconversion as the dependent variable and DMT group as the independent variable, and with age, sex, disease duration, time interval to last DMT intake as well as absolute lymphocyte count (for S1PM and ATZ+CLA subgroups) or complete B‐cell depletion (for the CD20‐mAb subgroup) as covariates.

^c^ Reference category: incomplete B‐cell depletion.

In the S1PM subgroup, the likelihood of seroconversion increased with higher lymphocyte count (OR 1.31 per 0.1 G/L), while in patients on alemtuzumab/cladribine, seroconversion was associated with time since last DMT intake (OR 1.38 per month) but not with lymphocyte count. In patients on CD20 mAbs, complete B‐cell depletion significantly decreased the probability of seroconversion (OR 0.52; *p* = 0.038), whereas time since last DMT intake did not. Sensitivity analyses did not indicate an aberrant effect for other DMTs.

**FIGURE 2:**
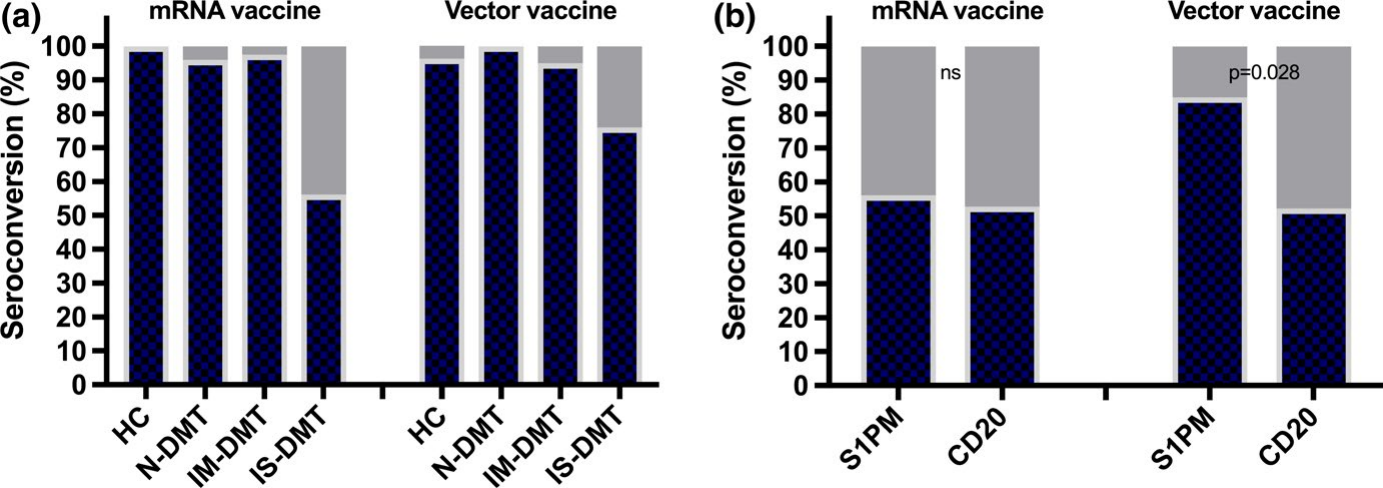
Seroconversion after SARS‐CoV‐2 vaccination depending on type of vaccine comparing healthy controls, N‐DMT IM‐DMT, IS‐DMT (panel A) as well as S1PM and CD20 (panel B). CD20, anti‐cluster of differentiation 20 monoclonal antibodies (ocrelizumab or rituximab); DMT, disease‐modifying treatment; IM‐DMT, immunomodulating DMT (dimethyl fumarate, glatiramer acetate, interferon‐beta preparations, natalizumab and teriflunomide); IS‐DMT, immunosuppressive DMT (alemtuzumab, cladribine, fingolimod, ocrelizumab, ozanimod, siponimod or rituximab); N‐DMT, no DMT (untreated); S1PM, sphingosine 1 receptor modulators (fingolimod, ozanimod, siponimod) [Colour figure can be viewed at wileyonlinelibrary.com]

Comparing rates of seroconversion between mRNA and vector‐based vaccines, there were no significant differences among the HC (100% vs. 96.3%), N‐DMT (96% vs. 100%) or IM‐DMT groups (97.5% vs. 95%). However, 56.2% of patients (103/183) on IS‐DMTs receiving an mRNA vaccination displayed seroconversion as opposed to 76.7% of those on vector‐based vaccines (33/43; *p* < 0.001 [Figure 2a]). Focusing on the IS‐DMT group, seroconversion after mRNA vaccination did not differ between S1PM and CD20‐mAb treatment (56.1% [32/57] vs. 52.7% [49/93]; *p* = 0.737), but after vector‐based vaccination the S1PM group had seroconversion significantly more often than the CD20‐mAb group (85% [17/20] vs. 52.2% [12/23]; *p* = 0.028 [Figure 2b]).

Neither the rate of any adverse events (36–39%), nor local (18–20%) and systemic adverse events (8–12%) differed between the groups. Only two severe adverse events occurred, both allergic reactions (one in the HC group and one in the N‐DMT group), which resolved without residual symptoms. Three patients reported SARS‐CoV‐2 infection (one patient each in the HC, N‐DMT and IS‐DMT groups), with one asymptomatic course and two patients experiencing mild COVID‐19 symptoms. There were no severe courses, no hospitalizations, no intensive care unit admissions, and no deaths.

## DISCUSSION

The rate of seroconversion after SARS‐CoV‐2 vaccination is excellent (>96%) in patients with MS who are untreated or who receive IM‐DMTs, and antibody levels do not differ from those of HCs. However, IS‐DMTs are associated with a lower probability of seroconversion; in patients on alemtuzumab/cladribine, vaccine response is decreased only slightly, and, unsurprisingly, this is dependent on the interval to last administration. Seroconversion is reduced under anti‐CD20‐mAb or S1PM treatment (53% and 64%, respectively), but protective humoral response is still expected in the majority of patients.

These results contradict a report of seroconversion rates of 1/26 (3.8%) in patients on fingolimod and 10/44 (22.7%) in those on ocrelizumab, but are in line with other more recently published reports of seroconversion rates ranging between 39% and 72% in patients on anti‐CD20 mAbs and between 50% and 100% in those on S1PMs [4, 5, 6, 8, 9]. This discrepancy could be explained as a statistical outlier owing to the small sample size in the Israelian study. However, there might be other factors involved, depending on specific DMT substances.

In patients on S1PMs, an important factor influencing humoral response is likely the degree of lymphopenia, which was unusually frequent in the Israelian study (88%) and was a significant predictor of seroconversion (OR 1.3 per 0.1 G/L) in our cohort [4]. In the anti‐CD20‐mAb group, degree of B‐cell depletion, which was not reported in the Israelian study, was a strong predictor of humoral response, in line with other recent studies [4, 10, 11, 12]. Of note, the predictive value of time from last infusion was obliterated by B‐cell depletion when analyzed together in a multivariable model, whereas other studies identifying time from last infusion as a predictor of seroconversion did not correct for the number of B cells [4, 8, 10]. Hence, B‐cell repletion is likely more important than the absolute time window for the probability of seroconversion. While monitoring of B‐cell reconstitution might aid in estimating chances of immune response to vaccination, extending dosing intervals is currently not recommended due to the potential risk of disease reactivation [13]. In this context, it is paramount to point out that even patients with complete peripheral B‐cell depletion (<1%) can develop humoral response and that patients with a lack of humoral response can still generate a robust T‐cell response, which plays an important role in the immune response to SARS‐CoV‐2 [9, 10].

It is not therefore warranted to withhold the opportunity for potential protection from SARS‐CoV‐2 by vaccination from any MS patients, independent of DMT or lymphocyte status.

Curiously, currently available data suggest a discrepancy in patients on S1PMs between the rates of seroconversion after SARS‐CoV‐2 infection (69–100%) and SARS‐CoV‐2 mRNA vaccination [14, 15, 16]. While this may simply represent an outlier explained by a small sample size, immunological response to SARS‐CoV‐2 infection and vaccination may differ under the influence of S1PMs [17]. Our study hints towards vaccine type playing a role because seroconversion was observed only in 56% of patients on S1PMs after mRNA vaccination but in 85% after vector‐based vaccination, while no difference was detected for CD20 mAbs or any other DMT. This could well be attributable to the limited sample size, but it is worth considering specific immunological effects of S1PMs on response to mRNA vaccination. Levels of secreted cytokines and efficacy of lymphocyte egress could vary, the adenovirus vector could amplify the immune response or S1PMs may interact with the pharmacodynamics of mRNA vaccines [17]. While the optimal vaccination strategy in immunocompromised patients is unclear, vector‐based vaccines could achieve a higher rate of humoral response in patients on S1PM treatment. This possible advantage would have to be balanced against a potentially more unfavorable safety profile, particularly the risk of vaccine‐induced thrombotic thrombocytopenia.

Some limitations of this study should be acknowledged. The study was powered for comparison of the four DMT groups on humoral response but not for comparisons among each individual DMT substance. However, power issues were mitigated by grouping DMTs with similar degree of impact on expected vaccine response, as well as by conducting predefined subgroup analyses comparing the substances of particular interest to the cohort. In addition, we did not assess T‐cell responses, which likely contribute to vaccine efficacy [10].

In conclusion, SARS‐CoV2 vaccination is safe in MS patients and humoral response is generally excellent. While reduced by IM‐DMTs, most importantly by B‐cell‐depleting CD20 mAbs and S1PMs, protective humoral response is still to be expected in the majority of patients.

In contrast to previously formulated opinions, we therefore firmly advocate that, while timing of vaccination may be individually tailored depending on a‐priori risk and DMT status, SARS‐CoV2 vaccination should be offered to every MS patient.

## CONFLICT OF INTEREST

Gabriel Bsteh has participated in meetings sponsored by, received speaker honoraria or travel funding from Biogen, Celgene/BMS, Lilly, Merck, Novartis, Roche, Sanofi‐Genzyme and Teva, and received honoraria for consulting for Biogen, Celgene/BMS, Novartis, Roche, Sanofi‐Genzyme and Teva. Harald Hegen has participated in meetings sponsored by, and received speaker honoraria or travel funding from Bayer, Biogen, Merck, Novartis, Roche, Sanofi‐Genzyme, Siemens and Teva, and received honoraria for consulting for Biogen, Novartis and Teva. Gerhard Traxler has participated in meetings sponsored by, and received honoraria (lectures, advisory boards, consultations) or travel funding from Biogen, Celgene/BMS, Merck, Novartis, Roche, Sanofi‐Genzyme and Teva. Nik Krajnc has participated in meetings sponsored by and received speaker honoraria or travel funding from Roche, Novartis and Merck, and has received a grant for a Multiple Sclerosis Clinical Training Fellowship Program from ECTRIMS. Fritz Leutmezer has participated in meetings sponsored by or received honoraria for acting as an advisor/speaker for Bayer, Biogen, Celgene/BMS, MedDay, Merck, Novartis, Roche, Sanofi‐Genzyme and Teva. Franziska Di Pauli has participated in meetings sponsored by and received honoraria (lectures, advisory boards, consultations) or travel funding from Bayer, Biogen, Celgene/BMS, Merck, Novartis, Sanofi‐Genzyme, Roche and Teva. Barbara Kornek has received honoraria for speaking and for consulting from Biogen, BMS‐Celgene, Johnson&Johnson, Merck, Novartis, Roche, Teva and Sanofi‐Genzyme, outside of the submitted work, and has no conflict of interest with respect to the present study. Paulus Rommer has received honoraria for consultancy/speaking from AbbVie, Allmiral, Alexion, Biogen, Merck, Novartis, Roche, Sandoz, Sanofi Genzyme, and research grants from Amicus, Biogen, Merck and Roche. Gudrun Zulehner has participated in meetings sponsored by or received travel funding from Biogen, Merck, Novartis, Roche, Sanofi‐Genzyme and Teva. Sophie Dürauer, Angelika Bauer, Sarah Kratzwald, Sigrid Klotz and Michael Winklehner have nothing to disclose. Florian Deisenhammer has participated in meetings sponsored by or received honoraria for acting as an advisor/speaker for Alexion, Almirall, Biogen, Celgene, Merck, Novartis, Roche and Sanofi‐Genzyme. His institution received scientific grants from Biogen and Sanofi‐Genzyme. Michael Guger has received support and honoraria for research, consultation, lectures and education from Almirall, Bayer, Biogen, Celgene/BMS, Janssen, MedDay, Merck, Novartis, Octapharma, Roche, Sanofi‐Genzyme, Shire and Teva. Romana Höftberger has received honoraria for lectures from Novartis and Biogen. Thomas Berger has participated in meetings sponsored by and received honoraria (lectures, advisory boards, consultations) from pharmaceutical companies marketing treatments for MS: Allergan, Bayer, Biogen, Bionorica, Celgene/BMS, GSK, Janssen‐Cilag, MedDay, Merck, Novartis, Octapharma, Roche, Sanofi‐Genzyme and Teva. His institution has received financial support in the past 12 months by unrestricted research grants (Bayer, Biogen, Celgene/BMS, Merck, Novartis, Sanofi Aventis, Teva) and for participation in clinical trials in MS sponsored by Alexion, Bayer, Biogen, Celgene/BMS, Merck, Novartis, Octapharma, Roche, Sanofi‐Genzyme and Teva.

## AUTHOR CONTRIBUTIONS

**Gabriel Bsteh:** Conceptualization (lead); Data curation (equal); Formal analysis (lead); Methodology (equal); Writing – original draft (lead). **Harald Hegen:** Conceptualization (supporting); Data curation (supporting); Investigation (supporting); Writing – review and editing (supporting). **Gerhard Traxler:** Data curation (equal); Methodology (equal); Writing – review and editing (equal). **Nik Krajnc:** Data curation (lead); Methodology (equal); Writing – review and editing (equal). **Fritz Leutmezer:** Methodology (equal); Writing – review and editing (equal). **Franziska Di Pauli:** Methodology (equal); Writing – review and editing (equal). **Barbara Kornek:** Methodology (equal); Writing – review and editing (equal). **Paulus Stefan Rommer:** Methodology (equal); Writing – review and editing (equal). **Gudrun Zulehner:** Methodology (equal); Writing – original draft (equal). **Sophie Duerauer:** Methodology (equal); Writing – review and editing (equal). **Angelika Bauer:** Methodology (equal); Writing – review and editing (equal). **Sarah Kratzwald:** Methodology (equal); Writing – review and editing (equal). **Michael Winklehner:** Methodology (supporting); Writing – review and editing (equal). **Sigrid Klotz:** Methodology (supporting); Writing – review and editing (equal). **Florian Deisenhammer:** Methodology (equal); Supervision (supporting); Writing – review and editing (equal). **Michael Guger:** Methodology (equal); Supervision (supporting); Writing – review and editing (equal). **Romana Hoeftberger:** Conceptualization (supporting); Methodology (equal); Supervision (supporting); Writing – review and editing (equal). **Thomas Berger:** Conceptualization (supporting); Methodology (equal); Supervision (equal); Writing – review and editing (equal).

## Data Availability

Data supporting the findings of this study are available from the corresponding author upon reasonable request and upon approval by the ethics committee of the Medical University Vienna.
